# Hybrid nanostructures for neurodegenerative disease theranostics: the art in the combination of biomembrane and non-biomembrane nanostructures

**DOI:** 10.1186/s40035-024-00436-7

**Published:** 2024-08-27

**Authors:** Chao Gao, Ran Xiong, Zhi-yu Zhang, Hua Peng, Yuan-kai Gu, Wei Xu, Wei-ting Yang, Yan Liu, Jie Gao, You Yin

**Affiliations:** 1 Department of Neurology, Second Affiliated Hospital (Shanghai Changzheng Hospital) of Naval Medical University, Shanghai, 200003 China; 2 Department of Neurology, Shanghai East Hospital, School of Medicine, Tongji University, Shanghai, 200120 China; 3 Department of Health Management, Second Affliated Hospital (Shanghai Changzheng Hospital) of Naval Medical University, Shanghai, 200003 China; 4 Department of Clinical Pharmacy, Xinhua Hospital, Clinical Pharmacy Innovation Institute, Shanghai Jiao Tong University School of Medicine, Shanghai, 200000 China; 5 Clinical Pharmacy Innovation Institute, Shanghai Jiao Tong University School of Medicine, Shanghai, 200092 China; 6 Changhai Clinical Research Unit, Shanghai Changhai Hospital, Naval Medical University, Shanghai, 200433 China; 7 Shanghai Key Laboratory of Nautical Medicine and Translation of Drugs and Medical Devices, Shanghai, 200433 China

**Keywords:** Neurodegenerative diseases, Biomembrane, Hybrid nanostructure, Alzheimer's disease, Parkinson's disease, Diagnosis

## Abstract

The diagnosis of neurodegenerative diseases (NDDs) remains challenging, and existing therapeutic approaches demonstrate little efficacy. NDD drug delivery can be achieved through the utilization of nanostructures, hence enabling multimodal NDD theranostics. Nevertheless, both biomembrane and non-biomembrane nanostructures possess intrinsic shortcomings that must be addressed by hybridization to create novel nanostructures with versatile applications in NDD theranostics. Hybrid nanostructures display improved biocompatibility, inherent targeting capabilities, intelligent responsiveness, and controlled drug release. This paper provides a concise overview of the latest developments in hybrid nanostructures for NDD theranostics and emphasizes various engineering methodologies for the integration of diverse nanostructures, including liposomes, exosomes, cell membranes, and non-biomembrane nanostructures such as polymers, metals, and hydrogels. The use of a combination technique can significantly augment the precision, intelligence, and efficacy of hybrid nanostructures, therefore functioning as a more robust theranostic approach for NDDs. This paper also addresses the issues that arise in the therapeutic translation of hybrid nanostructures and explores potential future prospects in this field.

## Introduction

Neurodegenerative diseases (NDDs) are a diverse group of neurological disorders characterized by the progressive loss of neurons in the peripheral nervous system or the central nervous system (CNS), leading to cognitive and behavioral deficits [1]. These diseases include amyotrophic lateral sclerosis, Alzheimer's disease (AD), multiple sclerosis (MS), Parkinson's disease (PD), and Huntington's disease (HD). The prevalence of NDDs is on the rise due to the aging of the population. The World Health Organization has projected that the number of individuals with NDDs will triple over the next 30 years [1, 2].

**Fig. 1:**
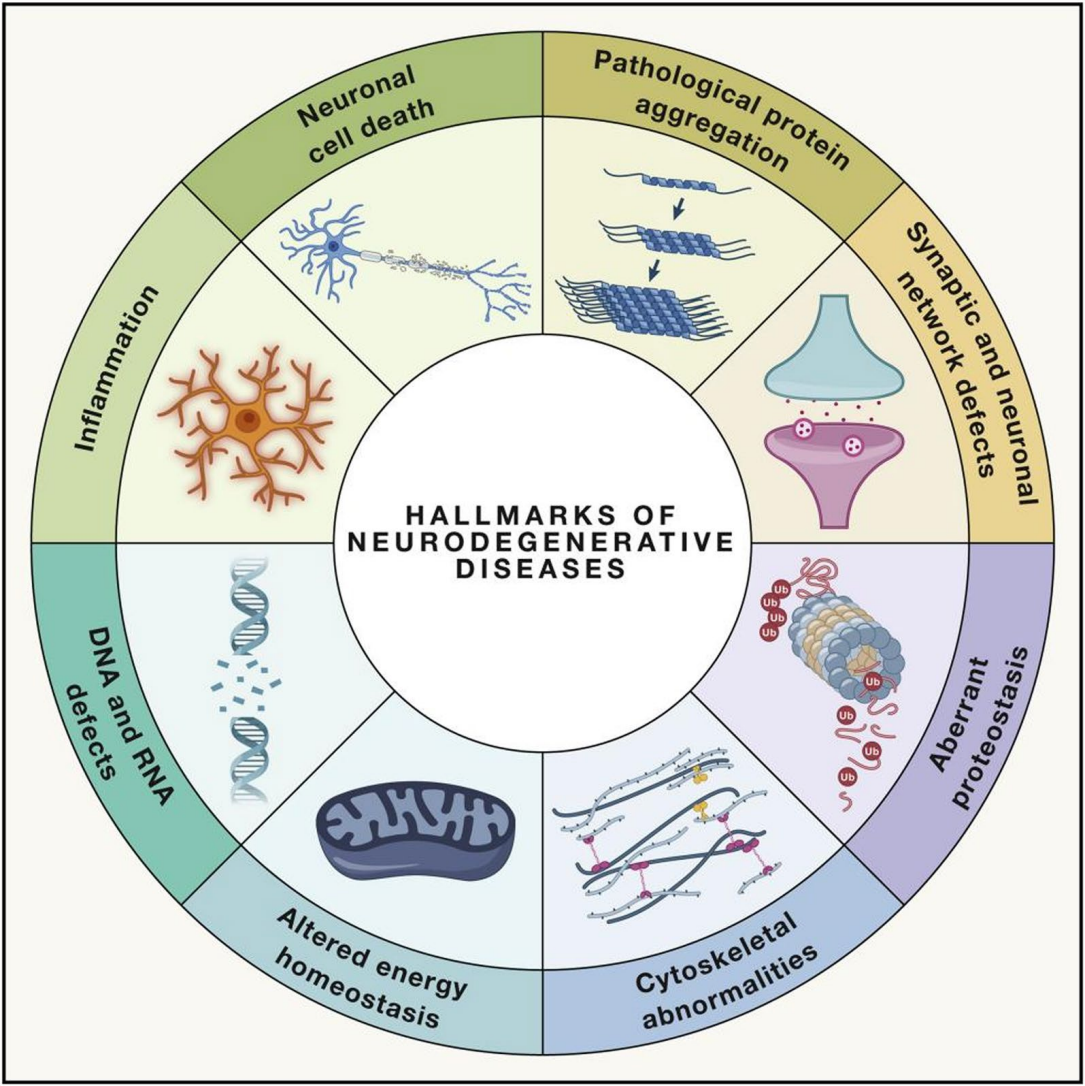
Hallmarks of NDDs. NDDs are a group of disorders that have several common characteristics. Image adapted from David M Wilson 3rd. Cell. 2023 [4] with permission from Elsevier Inc. Copyright 2022

Although each NDD has distinct characteristics, several common features are observed across these disorders [3]. These include pathological protein aggregation, dysfunction of synaptic and neuronal networks, abnormal proteostasis, cytoskeleton abnormality, changes in energy metabolism, defects in DNA and RNA, inflammation, and neuronal cell death (Fig. 1) [4]. The clinical hallmark of NDDs, protein aggregation, aids in the diagnosis of the condition [5–7]. The spread of neurodegeneration between cells and brain regions is mainly attributed to the misfolding of pathological proteins and the quick spread of their aggregates [8–11]. Although non-protein NDDs also exist, mature protein aggregates are the primary cause of the illness [12]. Pathologic protein aggregation can occur inside synapses and affect synaptic functions [13]. This usually leads to a kind of confusion in the neural network [14]. The resultant aberrant neuronal death, impaired energy metabolism, oxidative stress, protein degradation, and cytoskeletal abnormalities are directly linked to synaptic dysfunction [15–17]. As a result, numerous hallmarks are associated with and involved in NDDs, necessitating the use of multi-targeting therapeutics.

Despite the extensive research efforts dedicated to studying these disorders, current interventional approaches can only ameliorate symptoms rather than halting the progression of NDDs [18]. Hence, there is a pressing need for treatments that might significantly influence the progression of neurodegeneration. Multiple pharmaceuticals are now being tested in clinical trials for NDDs; however, a significant number of them has been discontinued due to inadequate effectiveness or substantial side effects. Hence, to mitigate the adverse effects of these traditional treatments, innovative therapeutic approaches are needed to achieve successful management of NDDs.

**Fig. 2:**
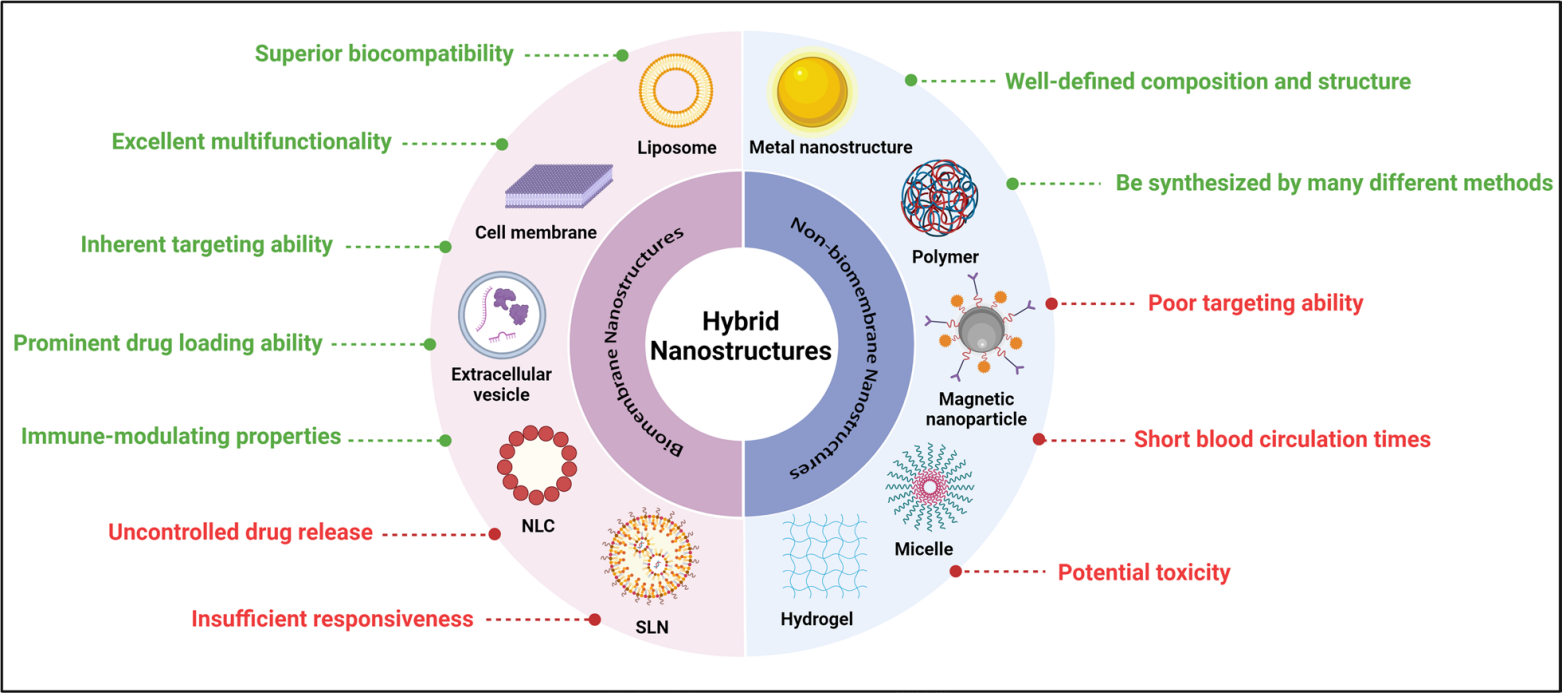
Advantages and drawbacks of biomembrane nanostructures and non-biomembrane nanostructures. The color green symbolizes the benefits of hybrid nanostructures, while the red color stands for their drawbacks. Image created with Biorender.com

Neuronanotechnology is an innovative method to circumvent obstacles in delivering drugs to the brain. Nanoparticles (NPs) possess distinctive characteristics, including customized size, shape, flexibility, and surface charge, which permit enhancement of therapeutic bioavailability and cellular uptake, reduction of drug resistance, and mitigation of side effects [19]. Consequently, these materials can serve as suitable vehicles for the diagnosis and treatment of NDDs. Various nanomaterial platforms, such as polymer-based NPs, dendrimers, lipid-based NPs, hydrogels, cyclodextrins, carbon-based NPs, and metal-based NPs, are utilized for therapeutic delivery to the brain [20]. There are several advantages associated with this approach. First, it allows for the controlled drug release at a predetermined rate. Second, NPs may be delivered either locally or systemically. Third, the NPs can remain in the body for a specified duration. Last, this approach enables targeted delivery of drugs to the precise site of action [21]. There are two primary categories of nanostructures that have been developed for nanomedicine applications: biomembrane nanostructures and non-biomembrane nanostructures (Fig. 2).

Biomembrane nanostructures are formed from biomembranes, including liposomes, cell membranes, extracellular vesicles (EVs), nanostructured lipid carriers (NLCs), and solid lipid nanoparticles (SLNs). These nanostructures possess notable advantages in biocompatibility, multifunctionality, and inherent targeting capability. Furthermore, these nanostructures have the ability to encapsulate both hydrophilic and hydrophobic medications, hence greatly enhancing their versatility [22]. Nevertheless, the use of biomembrane nanostructures is significantly restricted due to the unregulated drug release and inadequate responsiveness. These restrictions can be overcome by modifying the surface and using targeted ligands. Non-biomembrane nanostructures are commonly used in drug carriers, including metal nanostructures, polymers, magnetic NPs, micelles, and hydrogels, due to their distinctive physical and chemical characteristics. The non-biomembrane nanostructures have substantial specific surface area and their surface morphology can be modified [23–25]. Moreover, they can be fabricated  by many techniques, and the metal nanostructures have shown therapeutic effects on animal models of NDDs [26, 27]. Nevertheless, the non-biomembrane nanostructures also have side effects. They may induce significant harmful effects on healthy cells [28]. Furthermore, nanostructures that are not based on biomembranes can be readily eliminated by the reticuloendothelial system (RES), exhibit brief blood circulation durations, and may accumulate in healthy organs such as the liver and spleen, resulting in diminished therapeutic effectiveness [29]. Consequently, it is imperative to enhance the capacity of non-biomembrane nanostructures to target NDDs and mitigate their side effects [30]. This can be achieved by producing hybrid nanostructures.

The hybrid nanostructures have following advantages: (1) long circulation time by reducing nonspecific phagocytosis and RES clearance [31]; (2) good biocompatibility achieved through modifications in the composition, shape, and surface chemical characteristics of the nanostructures [32]; (3) specific targeting through mechanisms that underlie the inherent targeting of biomembranes or ligand changes; and (4) potent drug-carrying ability for both hydrosoluble and hydrophobic medicines.

**Fig. 3:**
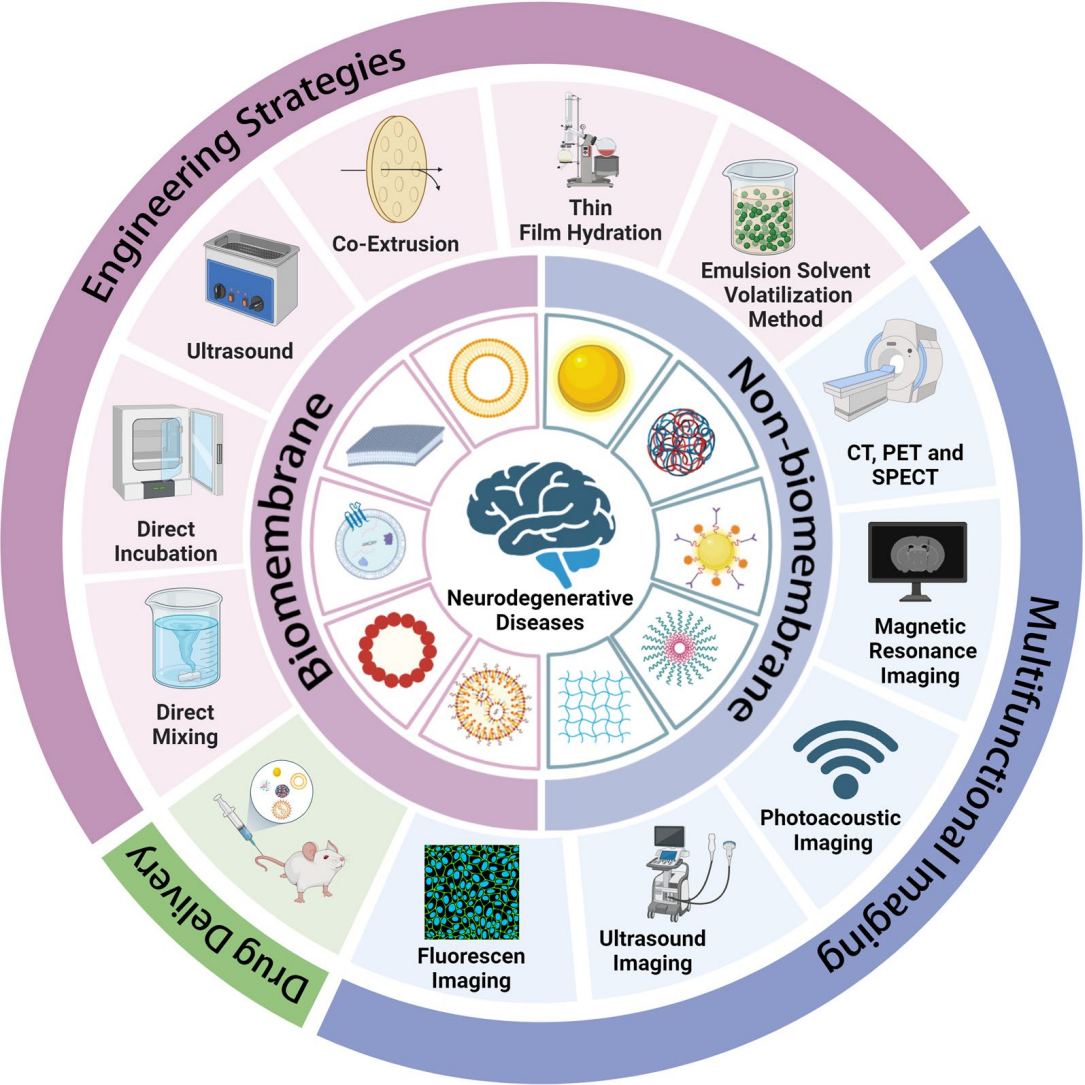
An overview of the use of hybrid nanostructures in the detection and management of NDDs, covering drug delivery, engineering approaches for synthesis, and diagnostic imaging. Image created with Biorender.com

There is a scarcity of literature examining the application of hybrid nanostructures for the treatment of NDDs in animal models. In this paper, we provide a comprehensive review of the engineering strategies for hybrid nanostructures, as well as the properties and applications of hybrid nanostructures in the field of NDD theranostics (Fig. 3).

## Engineering strategies for hybrid nanostructures

The primary goal of hybrid methodologies is to enhance the targeting precision, the biosafety, and the drug delivery capabilities of nanocarriers while preserving the integrity of the blood–brain barrier (BBB) [33].

Liposomes are the most extensively utilized nanostructures in biomembrane technology. Natural biomembrane-based nanostructures, known as cell membrane-based nanostructures, have a distinctive biomimetic design and can encapsulate various nanocores for the purpose of treating NDDs. EVs are minute membrane vesicles that are released by nearly all live cells. They have the ability to traverse diverse biological obstacles to access specific cells. The SLN is an advanced delivery technology that consists of a solid lipid core and a biocompatible surfactant outer shell. SLN has several distinctive advantages, including a notable drug loading capacity, compact dimensions, increased effective surface area, superior stability compared to alternative lipid carriers, biocompatibility, compatibility with a diverse array of therapeutics, minimal toxicity profile, and enhanced solubility and permeability. The nanostructures SLN and NLC exhibit notable similarities and possess a greater loading capacity [34]. In contrast, metal nanostructures have gained considerable interest in the realm of non-biomembrane nanostructures because of their distinctive characteristics. Metal nanostructures can be disseminated or contained within metal shells and covalently linked to their surfaces, facilitating effective delivery of medicinal medications to the brain. Polymeric NPs have been extensively studied for the purpose of delivering a wide array of treatments to the brain, including tiny hydrophilic molecules, lipophilic medications, proteins, peptides, and several other macromolecules [35]. The majority of current magnetic NPs have demonstrated nontoxic properties and exhibited favorable absorption capacities. Additionally, magnetic NPs have the potential to be used as diagnostic instruments [36]. Micelles possess a hydrophilic shell and a hydrophobic core, facilitating the capture of hydrophobic molecules inside the core and hydrophilic components within the shell [37]. Surface modifications with target active ligands can facilitate medication transport to the intended site while decreasing off-site distribution [38]. Hydrogels possess numerous advantages, including controlled drug release, degradability, biocompatibility, and stable mechanical strength [39, 40]. Therapeutic medications can be released in a continuous and gradual manner throughout the body, resulting in long-term disease therapy [41].

**Fig. 4:**
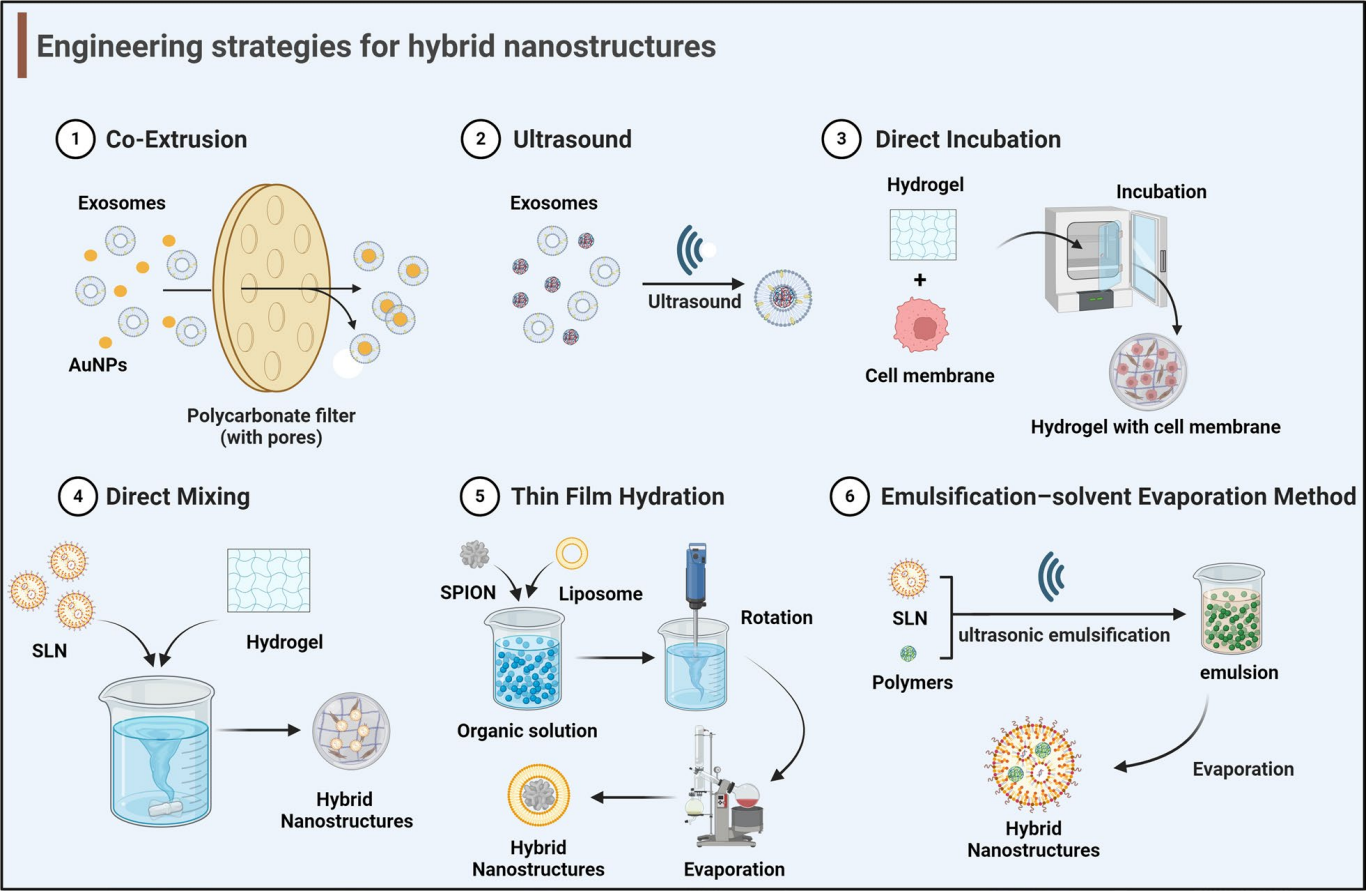
Six engineering strategies for hybrid nanostructures, including (1) co-extrusion, (2) ultrasound, (3) direct incubation, (4) direct mixing, (5) thin film hydration and (6) emulsification-solvent evaporation method. Image created with Biorender.com

As stated above, both biomembrane nanostructures and non-biomembrane nanostructures possess inherent limitations. Hybrid nanostructures can address the limitations of both methods by enhancing the permeability of the BBB and the bioavailability of drugs. Various methods can be used to achieve hybridization (Fig. 4).

### Co-extrusion

The most widely used method for producing synthetic liposomes is co-extrusion, usually referred to as physical extrusion [26]. The biomembrane and non-biomembrane nanostructure suspensions are combined in a specific ratio during the co-extrusion process. A porous filter membrane of the extruder with an appropriate size of pores was used to co-extrude the combined suspension several times [42]. After extrusion, hybrid nanostructures are generated by membrane wrapping of the nano core [43]. In the study by Khongkow et al., for instance, extrusion was utilized to hybridize gold NPs and exosomes, and the resulting hybrid nanostructures showed successful brain targeting [44]. Moreover, studies have been conducted on hybrid manganese dioxide NPs made from macrophage membranes using the co-extrusion method [45]. The co-extrusion process is straightforward and easy to conduct, and can fabricate hybrid nanostructures of the desired size by modifying the pore size of the filter membrane [46]. However, this method causes a significant waste of resources due to the inability to guarantee complete encapsulation of non-biomembrane nanostructures into biomembrane nanostructures.

### Ultrasound

Ultrasound treatment of a mixture of non-biomembrane and biomembrane nanostructures leads to the spontaneous generation of hybrid nanostructures [47–49]. Currently, hybrid nanostructures produced using this method have been tested in animal models of NDD. For example, superparamagnetic iron oxide nanoparticles (SPIONs), which are hybrid structures made of exosomes and polymers, have the ability to transport medications to the brain and improve the effectiveness of treatment [27, 50]. Compared to the co-extrusion method, the ultrasonic method is more simple to operate and cause less material loss. On the other hand, the membrane coating caused by the ultrasonic approach might not be uniform, and the NPs will be broken during the long-term ultrasonic process [31].

### Direct incubation

Sonication and physical extrusion are time-consuming and require much work. These methods also have a risk of compromising the integrity of biomembrane proteins [51]. Direct incubation is an approach that avoids these drawbacks. Hybrid nanostructures can be fabricated by directly incubating non-biomembrane nanostructures with cells or biomembrane nanostructures. For instance, Kutchy et al. produced a hybrid nanostructure through direct cell incubation with ultrasminy superparamagnetic iron oxide followed by isolation using a qEV column [52]. Hydrogels [53, 54] or gold NPs [55] can be hybridized with biomembrane nanostructures, extracellular vesicles, or exosomes produced by cells to produce hybrid nanostructures that may be useful for the detection or treatment of NDDs.

### Direct mixing

The aforementioned techniques mostly target membrane-containing nanostructures. Hybrid nanostructures cannot be fabricated with non-biomembrane nanostructures using the aforementioned techniques. Under some circumstances, biomembrane nanostructures can be combined directly with non-biomembrane nanostructures to induce hybridization. To treat PD, Uppuluri et al. loaded drug-containing SLNs in a thermoresponsive Methyl Cellulose in situ gel, producing a hybrid structure, which was shown to increase the drug delivery efficiency [56]. Furthermore, in the study by Adnet et al., drug-loaded liposomes were combined with in situ gels to fabricate hybrid nanostructures that could be used to treat AD specifically [57].

### Thin film hydration

One of the conventional techniques for fabricating  hybrid nanostructures is the thin film hydration method. This procedure involves mixing lipids with an organic solvent and removing the organic solvent using rotary evaporation [58]. By hybridizing liposomes and SPIONs, Saesoo et al. developed a hybrid nanostructure that increases the drug delivery efficiency and BBB penetration [59]. This technique was further refined by Shi et al. and is known as the lipid thin film dehydration method [60]. Due to the use of hazardous chemical solvents, the process has potential toxicity, although it is relatively well established and the equipment is straightforward [58].

### Emulsification–solvent evaporation method

The emulsification–solvent evaporation method involves creating an emulsion by stirring or ultrasonic emulsification of the aqueous and the organic phases and then evaporating the organic phase to cause the dispersion phase to encapsulate the medication to create nanostructures [61]. For instance, Gomes et al. hybridized SLNs and polymers to create hybrid nanostructures that show improved ability to target the brain [62]. Although there are numerous variables influencing the properties of hybrid nanostructures throughout the fabrication process, this technique has advantages of easy preparation and high reproducibility [61].

### Other hybridizing methods

In addition to the techniques mentioned above, hybrid nanostructures can also be generated using techniques such as in situ packaging, freeze‒thaw/ultrasound, extrusion/ultrasound and stirring, and extrusion/electroporation [43].

## Applications in NDD diagnosis

In the early stages of NDDs, patients are usually asymptomatic, and imaging techniques may provide useful adjunctive information [63]. In advanced stages, the diagnosis of NDDs is largely based on clinical assessment and imaging. Patients usually go to hospitals with symptoms present for a long time. Therefore, early diagnosis and screening for NDDs is crucial. The diagnostic tools for NDDs are very complex, and early stages of various NDDs show many overlapping symptoms that are difficult to distinguish and differentiate.

**Fig. 5:**
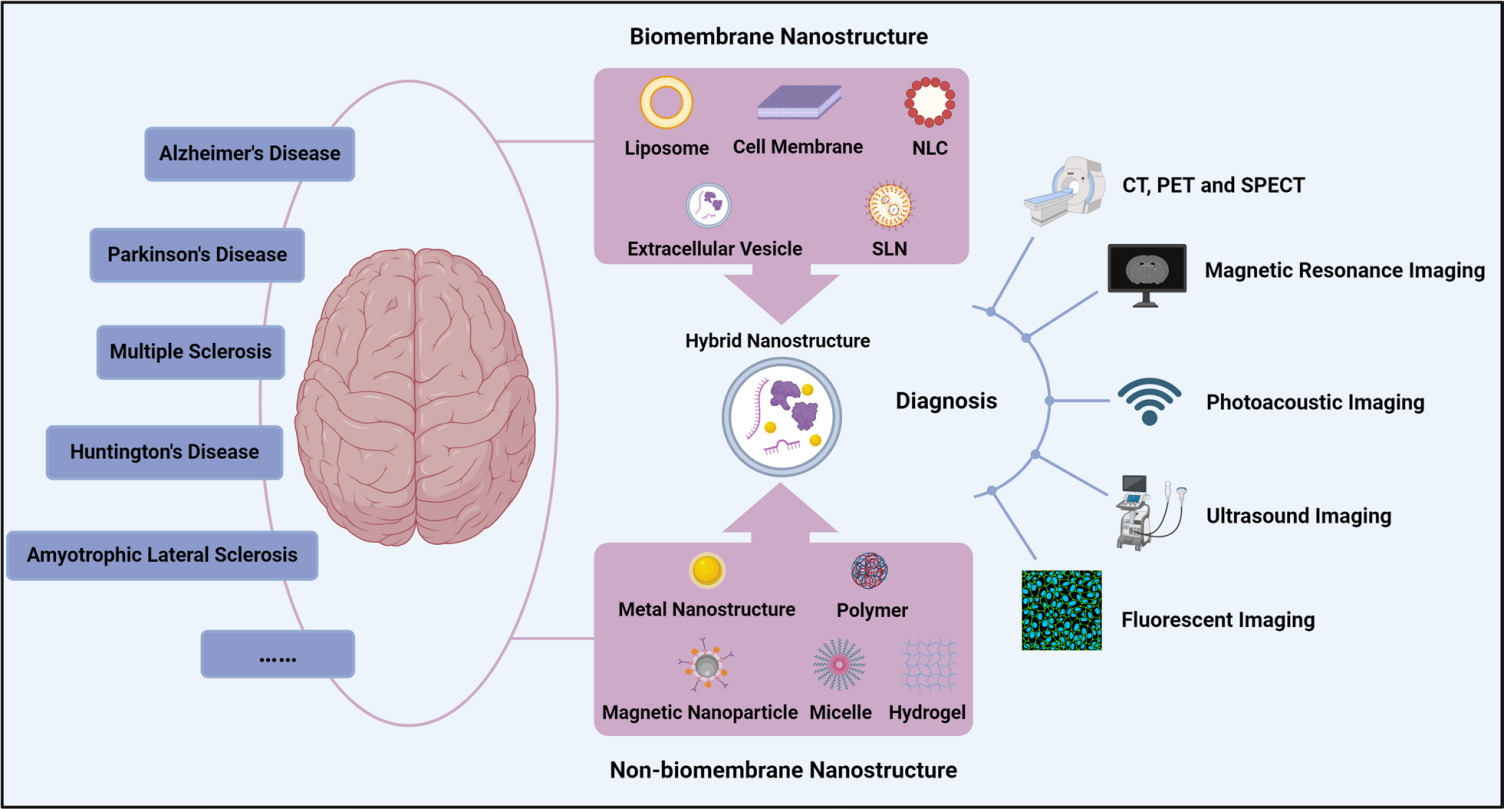
Hybrid nanostructures for the diagnosis of NDDs. Image created with Biorender.com

NPs are an important tool for molecular imaging involving multiple modes and functions. Superparamagnetic iron oxide, gold NPs/nanorods, manganese oxide (MnO), and quantum dots exhibit distinct characteristics, such as paramagnetism, surface plasmon resonance, superparamagnetism, and photoluminescence. These characteristics render them suitable for imaging, treatment, and drug delivery applications. Techniques used for targeted imaging include magnetic resonance imaging, positron emission tomography, single photon emission computed tomography, photoacoustic imaging, computed tomography (CT), two photon or fluorescent imaging, and ultrasound (Fig. 5) [64].

The main nanostructure hybridization approach investigated for the diagnosis of NDDs is the combination of non-biomembrane nanostructures with exosomes. Exosomes are membrane vesicles that originate from cells and have a size range of 40–100 nm [65]. Exosomes commonly consist of biological constituents derived from parental cells, including mRNAs, transport proteins, or proteins associated with distinct cellular functions [66]. Exosomes have the capacity to traverse biological barriers, such as the BBB, due to their signaling molecules, optimum size and membrane coating. Consequently, they may serve as a potent diagnostic tool [67]. However, there are still two major challenges in the field of exosome research: the ability to prepare exosomes consistently and the effectiveness of tracking exosomes in living organisms. Non-biomembrane nanostructures offer numerous benefits that can address the limitations of exosomes [68]. NPs coated with exosomal membranes have been used for imaging and treatment in animal models of NDDs. In a previous study, Au NPs were modified with neuron-targeted exosomes with the Lamp2b-rabies virus glycoprotein (RVG) and glycosylation-stabilized peptides on their surface [44]. Bioluminescent imaging of the mouse brain revealed that the Au NPs can traverse the BBB and specifically target brain cells both in vitro and in vivo. Compared with Au NPs coated with unmodified exosomes, the Au NPs coated with RVG-exosomes crossed the BBB more effectively and exhibited greater accumulation in brain cells. This study presents a promising method for overcoming the BBB obstacle and could lead to the development of efficient diagnostic techniques for NDDs [44]. CT imaging can be used to track exosomes loaded with Au NPs in vivo. For example, exosomes produced from bone marrow mesenchymal stem cells (MSCs) that were loaded with glucose-coated Au NPs were administered in murine models (AD and PD) through the intranasal route. Results showed that the exosomes specifically targeted and accumulated in pathologically relevant brain regions, with efficacy observed up to 96 h after administration [69].

Currently, the sole hybrid nanostructure for the diagnostic purposes of NDDs is composed of exosome-coated gold NPs. This hybridization technique has shown potential in enhancing disease diagnosis in experimental models. In future studies, more types of hybrid nanostructures should be explored for their applications in NDD diagnosis.

## Applications in NDD treatment

NDDs are difficult to treat. A major obstacle in the management of NDDs is the difficulty of medications to overcome the BBB barrier and selectively target neuronal cells [26]. The current therapeutics mostly aim to delay the progression of the diseases, rather than targeting the fundamental etiology [70]. Hybrid nanostructures, which involve the combination of biomembrane nanostructures with non-biomembrane nanostructures, show a substantial increase in BBB permeability and precise targeting of lesion sites in NDDs. This leads to major improvements in both diagnostic accuracy and therapeutic efficacy.

NDDs exhibit some common pathological characteristics, including neuronal loss, aberrant accumulation of protein aggregates, and persistent inflammation in specific brain areas [71]. Therefore, hybrid nanostructures are able to treat a wide range of NDDs by targeting these common pathological features. Li et al. designed macrophage-disguised MnO_2_ NPs loaded with fingolimod, and found that these NPs could consume reactive oxygen species (ROS) and produce oxygen (O_2_). Furthermore, the NPs can counteract the proinflammatory milieu by facilitating the phenotypic transformation of microglia via various signaling pathways, hence enhancing the protective effects on injured neurons [45]. Feng et al. developed a mesoporous Prussian blue nanozyme (MPBzyme@NCM) with a neutrophil-like cell-membrane (NCM) coating. They found that the MPBzyme@NCM can traverse the BBB and scavenge ROS when administered intravenously. Compared with both nondifferentiated HL-60 cell membrane coating and noncoated condition, the NCM coating resulted in sustained accumulation of MPBzyme@NCM in the damaged brain [72].

**Fig. 6:**
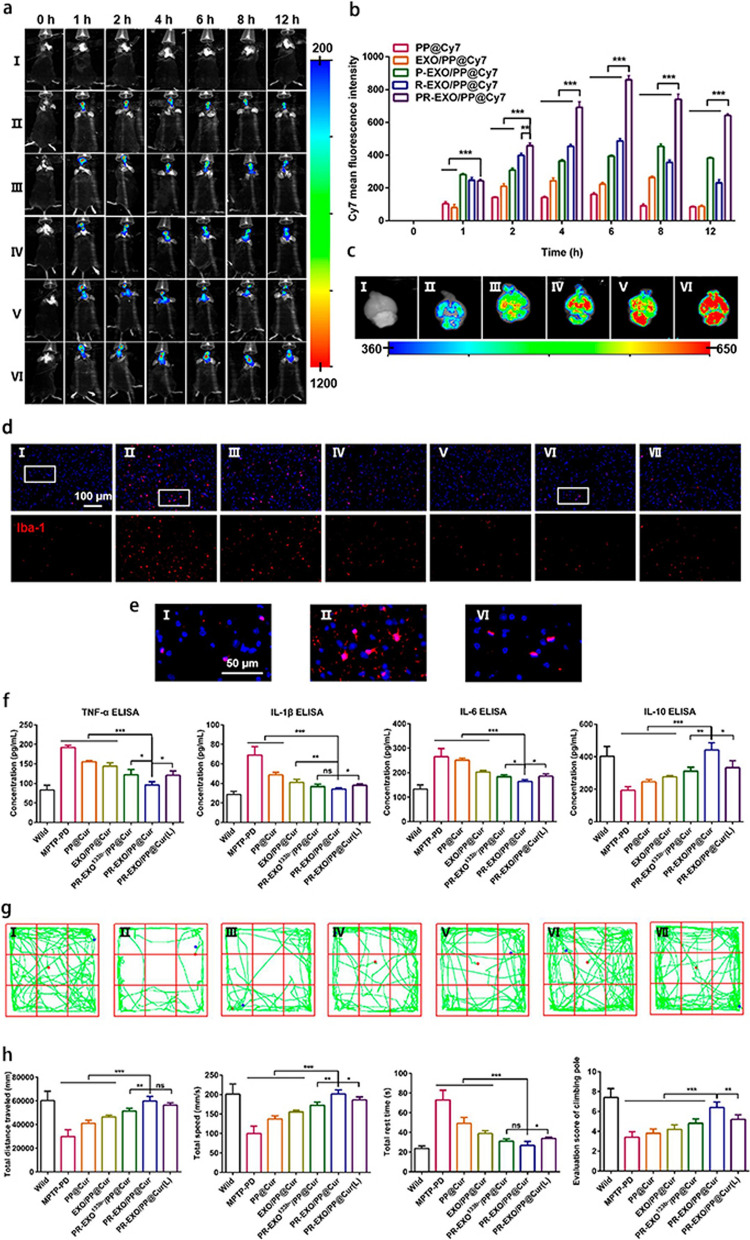
Biodistribution of drugs in the brain and therapeutic effects in vivo after nasal administration. **a** Fluorescence intensity of Cy7 in the brains of PD mice. **b** The mean fluorescence intensity of Cy7 in the mouse brain regions quantified from **a**. **c** Ex vivo biodistribution of different NPs in PD mouse brains after 6 h of nasal administration. Labels I–VI in panels **a** and **c** represent PBS, PP@Cur, EXO/PP@Cur, P-EXO/PP@Cur, R-EXO/PP@Cur, and PR-EXO/PP@Cur, respectively. Data are presented as the mean ± SD (*n* = 3). **d** Immunofluorescence staining of Iba-1 in the substantia nigra of wild-type and PD mice treated with different NPs. **e** Magnified images of microglia in wild-type mice (I), PD mice (II), and PD mice treated with the PR-EXO/PP@Cur nanocarriers (VI). **f** Serum concentrations of TNF-α, IL-1β, IL-6, and IL-10 in wild-type and PD mice treated with different NPs (*n* = 4). **g** Trajectories of wild-type and PD mice treated with different NPs in the open-field test. Labels I–VII in panels **d**, **e** and **g** represent Wild-type, MPTP-PD, PP@Cur, EXO/PP@Cur, PR-EXO133b–/PP@Cur, PR-EXO/PP@Cur, and PR-EXO/PP@Cur, respectively. **h** Total distance travelled, speed, and total resting time of wild-type and PD mice treated with different NPs in the open-field test. Scoring of wild-type and PD mice treated with different NPs in the pole-climbing test. Data are presented as the mean ± SD (*n* = 5). Student’s *t* test: *P < 0.05; **P < 0.01; ***P < 0.001; ns, not significantly different. Adapted from Peng H, et al. ACS Nano. 2022 [27] with permission from the American Chemical Society. Copyright 2022

Hydrogels are a three-dimensional cross-linked polymeric network that possesses a very hydrophilic structure [41]. Hydrogels have been extensively employed in the therapeutics of diverse diseases owing to their advantageous properties, including biodegradability, biocompatibility, robust mechanical integrity, and controlled release of drugs [39, 40]. Long-term neuroinflammation poses a significant barrier to neurological recovery in the context of neurodegenerative disorders. Zhang et al. developed a method to combine hydrogels with exosomes to deliver exosomes derived from interleukin-1β-stimulated bone marrow stromal cells (βExos) in a sustainable manner. This approach enhances exosome production and anti-inflammatory capabilities, leading to the inhibition of neuroinflammation and promotion of neurological recovery. Following in situ injection into the brain injury site, the released βExos effectively regulated neuroinflammation, resulting in considerable reductions of glial scar formation and neuronal loss [54]. Similarly, exosomes can be combined with SPIONs to create hybrid nanostructures that specifically target and treat animal models of NDDs. In the study by Peng et al. [27], the ROS-responsive polymer poly(propylene sulfide)-polyethylene glycol (PPS-PEG) were loaded with SPIONs and hydrophobic curcumin to form the micellar core (PP@Cur). The outer layer (PR-EXO) was formed by embedding SA-P (stearic acid-RQIKIWFQNRRMKWKK) and SA-RVG (stearylamine-YTIWMPENPRPGTPCDIFTNSRGKRASNG) into the phospholipid bilayer of exosomes. The self-oriented nanocarrier (PR-EXO/PP@Cur) was fabricated  by mixing the outer layer and the core and passing them through an extruder. After intranasal treatment, brain accumulation of PR-EXO/PP@Cur was observed within 12 h of nasal treatment (Fig. 6a, b). Furthermore, curcumin accumulation in the brain was detected ex vivo at 6 h after nasal administration of PR-EXO/PP@Cur (Fig. 6c). Compared to other treatment groups of MPTP (1-methyl-4-phenyl-1,2,3,6-tetrahydropyridine) PD mice, three weeks of PR-EXOs/PP@Cur treatment led to significantly decreased microglial infiltration in PD mice (Fig. 6d). Additionally, the administration of PR-EXOs/PP@Cur restored microglial morphology (Fig. 6e), decreased the concentrations of proinflammatory factors, such as TNF-α, interleukin (IL)-1β, and IL-6, while increasing the anti-inflammatory factor IL-10 in PD mice (Fig. 6f). Among the NPs tested, mice with PR-EXO/PP@Cur treatment exhibited the most pronounced enhancement in movement behavior and coordination ability (Fig. 6g, h) [27].

There are also studies focusing on the effects of hybrid nanostructures on specific pathological symptoms of a particular NDD [27, 50, 73, 74]. In the following, we will focus on three diseases: AD, PD, and MS.

### AD

AD is the prevailing type of dementia globally, with an estimated prevalence ranging from 50% to 80% of cases [75]. The etiology of AD is intricate, and its pathophysiology remains elusive. The main pathological characteristics include atypical accumulation of beta-amyloid protein (Aβ), aberrant phosphorylation of tau protein, and inflammation of the brain system, among other potential factors [76]. Currently, there are still few available therapies for AD.

One of the main treatments for AD that is being developed clinically is passive immunotherapy. Aβ peptide deposition is thought to be the primary initiator of neurodegenerative processes in AD [77]. Recent research shows that monoclonal antibodies can slow early AD progression and stimulate Aβ elimination from the brain. The antibodies bapineuzumab, solanezumab, crenezumab, gantenerumab, aducanumab, lecanemab, and donanemab have progressed the furthest in clinical development. The antibodies lecanemab, donanemab, and aducanumab can remove > 60% of Aβ deposits after 18 months of treatment, and have demonstrated efficacy in slowing clinical decline [78]. Lecanemab, however, was the first to obtain complete Food and Drug Administration (FDA) approval. Furthermore, only people with mild to moderate AD might benefit from monoclonal antibody medications in terms of cognitive performance. During treatment, doctors must be aware of the possible side effects including cerebral edema [79]. Researchers have investigated the potential therapeutic benefits of hybrid nanostructures in AD.

Cell membrane-coated NPs have been extensively studied as a potential solution to address this issue. This approach enhances the overall efficiency of nano-drug delivery systems [43]. Tang et al. fabricated macrophage membrane-encapsulated, rapamycin-loaded 4-(hydroxymethyl) phenylboronic acid pinacol ester NPs that are responsive to ROS. They found that the biomimetic NPs could specifically target the ROS region and release pharmaceuticals, with enhanced efficacy in drug delivery [80]. While their study tested application of the biomimetic NPs in atherosclerosis, these results also imply potential for AD as ROS also increase in AD. Thus, studies of hybrid NPs for the treatment of AD are anticipated.

Due to the biocompatibility and slow release properties of hydrogels, Cunha et al. developed in situ thermosensitive nasal gels with hybridized NLCs for direct delivery of rivastigmine to the brain. This nanosystem showed adequate nasal mucoadhesion and sustained drug release, providing a promising new treatment strategy for advancing the management of AD [81]. Adnet et al. integrated the liposome and hydrogel methodologies to create a composite nanosystem for nasal AD treatment. The results showed increased drug residence time in the nasal cavity and regulated release of the drug [57]. In a recent study examining the capacity of NPs and exosomes to improve neurological illnesses, copper sulfide NPs and exosomes produced from MSCs were administered together in rats with cadmium (Cd)-induced neurological disorders. The combined treatment improved total antioxidant status, increased BDNF and NGF, and showed anti-inflammatory effects. Histological assessments demonstrated that the combined treatment reduced the deleterious impacts of Cd on brain tissue [82].

In summary, hybrid nanostructures increase drug loading capacity, extend drug circulation time, improve drug targeting, and achieve therapeutic effects in AD treatment. Nevertheless, this strategy has not been extensively studied for AD treatment, and most of them are still in the preclinical stage (Table 1).

**Table 1:** Summary of studies on hybrid nanostructures based on biomembrane and non-biomembrane nanostructures for the treatment of AD

Nanomedicine	Biomembrane nanostructure	Non-biomembrane nanostructure	Method of administration	Therapeutic effects	Research stage	Reference
Liposomes of API	Liposomes	Hydrogel	Intranasal administration	Prolong API release and increase API residence time	In vitro	[57]
MSC-Exo + CuSNPs	MSC-Exo	CuSNPs	Oral administration	Ameliorate the oxidative stress and inflammatory markers; protect neurons	In vivo	[82]
In situ gel of rivastigmine-loaded NLC	NLC	In situ gel	Intranasal administration	Increase the viscosity and the mucoadhesion; show prolonged drug release	In vitro	[81]

API, Active Pharmaceutical Ingredient; CuSNP, copper sulfide nanoparticle; MSC-Exo, exosomes derived from mesenchymal stem cells

### PD

PD remains untreatable. Current therapeutic strategies primarily aim to enhance dopaminergic signaling. These therapies include levodopa (*L*-dopa) that inhibits DOPA decarboxylase, inhibitors of catechol-O-methyltransferase, dopamine agonists, and inhibitors of the enzyme monoamine oxidase type B [83]. Regrettably, the aforementioned treatments solely alleviate symptoms without impeding the clinical and pathological progression of PD [84].

The advancement of hybridization technology offers a viable approach for the management of PD. Liu et al. fabricated a RVG-exosome (REXO)-coated gene-chem nanocomplex REXO-C/ANP/S, by hybridising exosome and polymer. This nanocomplex effectively concentrates medications at the action site of a target cell. At the cellular and animal levels, the functions of REXO-C/ANP/S in clearing α-syn aggregates through the BBB and facilitating membrane fusion have been verified. This delivery system achieved efficient administration of siRNA and chemical drugs and reduced α-syn aggregates in diseased dopaminergic neurons [50]. In another study, to ensure effective transportation of piribedil to the brain, the SLNs were encapsulated in a thermoresponsive methyl cellulose in situ gel (PBD-SLN-ISG) to delay mucociliary clearance after oral administration in rats. Pharmacokinetic experiments conducted in living organisms demonstrated that compared with simple intranasal suspensions of PBD (PBD-Susp), PBD-SLN-ISG resulted in a 4-fold increase in the PBD (AUC)_brain_ and a 2.3-fold decrease in PBD (C_max_)_plasma_. In addition, PBD-Susp showed limited direct absorption from the nose to the brain, while the optimized PBD-SLN-ISG demonstrated efficient direct nose-to-brain absorption [56].

### MS

MS is an immune-mediated inflammatory disease of the the CNS, characterized by the presence of inflammatory lesions, demyelinating plaques, and irreversible damage to axons as the disease progresses [85]. Relapsing–remitting MS is the most common MS phenotype and is characterized by recurring relapses and remissions of neurological symptoms in affected individuals [86]. The aim of MS treatment is to minimize relapses and slow the course of the disease [87]. Disease-modifying treatments (DMTs) are commonly employed in long-term management to provide therapeutic advantages by inhibiting or regulating immune function and inflammatory responses [87, 88]. Nevertheless, the existing DMTs currently demonstrate restricted efficacy in arresting the course of MS [89].

With the advent of stem cell therapies, Ferreira et al. employed hydrogels and liposomes as a means to fabricate hybrid nanostructures for the purpose of delivering MSCs in the context of MS treatment. The engineered hydrogel carrying MSCs was administered intracerebroventricularly in an experimental autoimmune encephalomyelitis rat model. Compared with cell suspensions, the formulated solution was more effective in decreasing the severity of the disease and achieving maximum clinical score. Therefore, the hybrid nanostructures may provide a promising avenue for addressing the challenges associated with existing therapeutic approaches in the treatment of MS [90].

### Others

NDDs include many diseases. We searched literature in PubMed and the Web of Science using the following keywords: (((((((((((Hybrid Nanostructures[Title]) OR (liposome[Title])) OR (cell membrane[Title])) OR (extracellular vesicle[Title])) OR (nanostructured lipid carriers[Title])) OR (solid lipid nanoparticles[Title])) OR (metal nanostructure[Title])) OR (polymer[Title])) OR (magnetic nanoparticle[Title])) OR (micelle[Title])) OR (hydrogel[Title])) AND (((((((((Neurodegenerative diseases[Title]) OR (Alzheimer disease[Title])) OR (Parkinson disease[Title])) OR (primary tauopathies[Title])) OR (frontotemporal dementia[Title])) OR (amyotrophic lateral sclerosis[Title])) OR (synucleinopathies[Title])) OR (Huntington disease[Title])) OR (related polyglutamine (polyQ) diseases[Title])). However, we found no studies using hybrid nanostructures as carriers for the treatment of other NDDs.

Cholesterol production is markedly reduced in animal models of HD, and this is closely correlated with both synaptic dysfunction and cognitive impairment [91]. One potential HD treatment is to increase the amount of cholesterol through effective delivery to the brain [92]. In the study by Tosi et al., polylactic-co-glycolic acid (PLGA) and cholesterol were mixed to generate a novel NP that could transfer peripheral cholesterol across the BBB. The conjugated g7-glycopeptide (H_2_N-Glyl-Phe-*D*-Thr-Gly-*L*-Phe-*L*-Leu-*L*-Ser[*O*-β-*D*-glucose]-CONH_2_) on the surface of the NPs promotes the passage of particles across the BBB in a non-invasive manner by producing BBB membrane curvature and subsequent endocytosis [93]. In a more recent study by the same research group, the cholesterol and PLGA hybrid NPs modified with g7 (g7-PLGA-cholesterol) were found to be taken up by neurons mainly through the clathrin-dependent mechanism, and the g7-glycopeptide had a major effect on the interaction between the NPs and the cells. However, if the passage through the BBB is mediated by receptors, the amount of NPs that could reach the brain would be lower due to the potential saturation of these receptors. In conclusion, this gives us optimism for HD therapies and the use of hybrid nanostructures in the treatment of NDDs, even though this NP is not a hybridization of biomembrane and non-biomembrane nanostructures [94]. Research on the benefits of hybrid nanostructures in medicine delivery is currently lacking. Next, it is imperative to achieve accurate treatment outcomes by regulating and targeting the pathological environment in a targeted manner.

## Conclusions and prospects

NDD diagnosis and treatment are challenging. Many patients missed timely treatment due to delayed diagnosis. In addition, the longer the treatment duration is, the more detrimental the treatment outcome will be [95]. The nanotechnology holds great potential in the field of NDDs. Nanostructures can effectively transport pharmaceuticals to the brain while preserving the integrity of the BBB, hence serving as a therapeutic platform. This article describes various hybrid approaches involving biomembrane and non-biomembrane nanostructures. These methodologies possess distinct merits and shortcomings. Hybrid nanostructures hold significant value in the field of diagnosis and treatment of NDDs. Currently, the primary focus of research on hybrid nanostructures for the treatment of NDDs lies in the areas of AD and PD. By employing certain modification techniques [26], hybrid biomembrane nanostructures and non-biomembrane nanostructures have demonstrated significant therapeutic efficacy in basic research on NDDs.

However, the hybrid technology is primarily confined to experimental research and has yet to reach clinical implementation. To achieve the desired performance, several modification techniques are needed [33], such as genetic engineering, covalent coupling, and non-covalent modifications. The modification approaches play a crucial role in enhancing the BBB penetration and the brain-targeting capabilities of hybrid nanostructures.

**Fig. 7:**
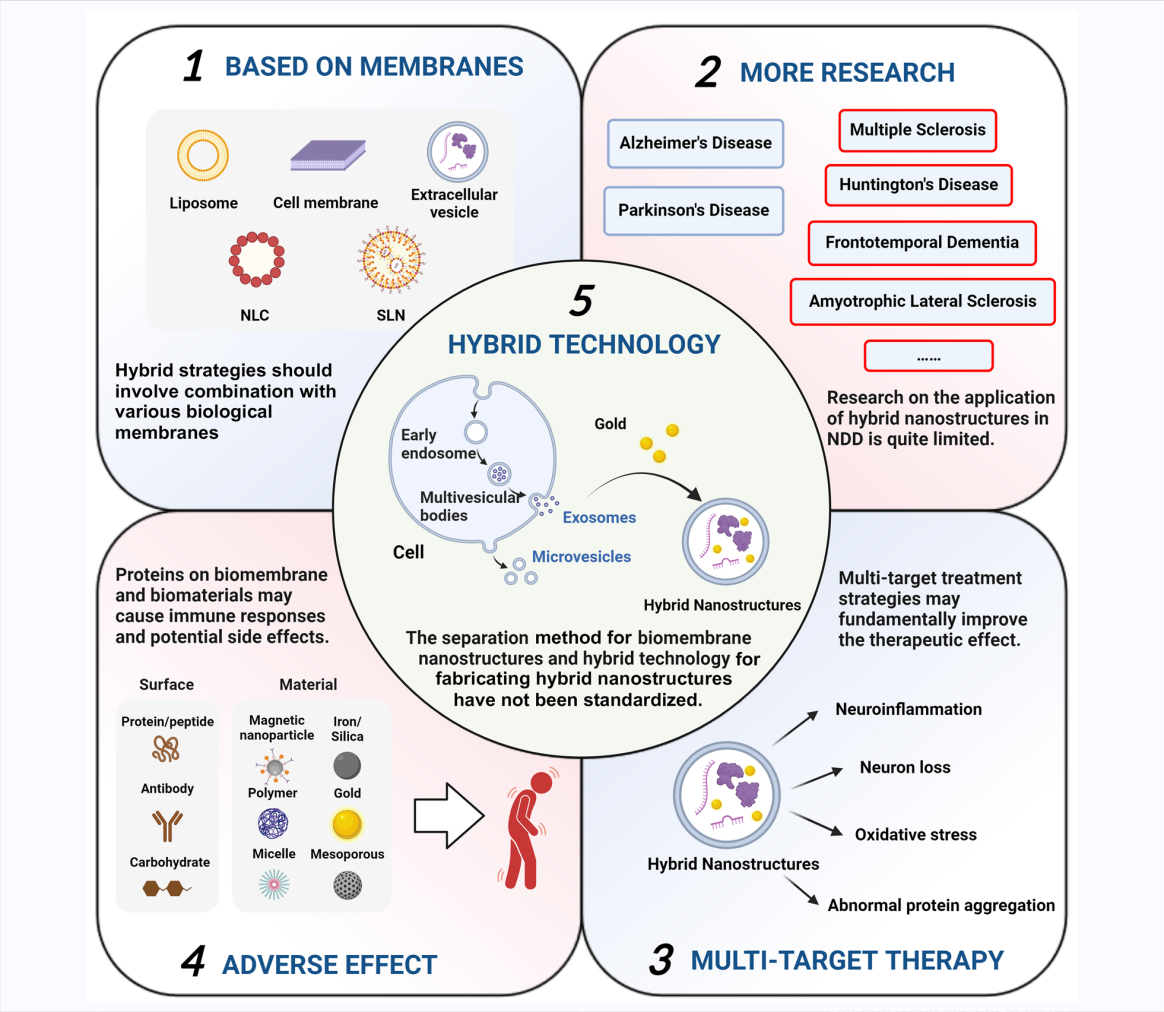
Challenges and perspectives of hybrid nanostructures for AD treatment. Image created with Biorender.com

Despite the promising advancements in hybrid nanostructures, there are several obstacles that must be addressed (Fig. 7). (1) The features of biological membrane nanostructures, such as cell membrane and exosomes, vary depending on the origin and the constituent membrane elements. Hybrid techniques that combine the distinct characteristics of different biological membranes are recommended. (2) Currently, studies of hybrid nanostructures for the treatment of NDDs in animal models are primarily focused on AD and PD. However, it is important to conduct further research on the treatment of other NDD diseases. (3) Many diseases are caused by more than one single pathological factor. Employing multi-target treatment strategies may significantly enhance therapeutic outcomes. (4) Proteins present on the biomembrane have the potential to elicit immunological responses. Biomaterials are present in non-biomembrane nanostructures. Further studies are warranted to examine the immunogenicity and potential side effects of biomembrane nanostructures before their clinical translation. (5) Additionally, standardization of the separation method for biomembrane nanostructures and the hybrid technology for fabricating hybrid nanostructures remains unresolved. Addressing these concerns may pave the way for therapeutic applications of hybrid nanostructures.

## Data Availability

Not applicable.

## Abbreviations

NDD: Neurodegenerative disease
CNS: Central nervous system
MS: Multiple sclerosis
AD: Alzheimer's disease
PD: Parkinson's disease
HD: Huntington's disease
NP: Nanoparticle
EV: Extracellular vesicle
NLC: Nanostructured lipid carrier
SLN: Solid lipid nanoparticle
RES: Reticuloendothelial system
BBB: Blood-brain barrier
SPION: Superparamagnetic iron oxide nanoparticle
MnO: Manganese oxide
CT: Computed tomography
RVG: Rabies virus glycoprotein
MSC: Mesenchymal stem cell
ROS: Reactive oxygen species
TNF-α: Tumor necrosis factor α
IL: Interleukin
MPTP: 1-Methyl-4-phenyl-1,2,3,6-tetrahydropyridine
Aβ: Beta-amyloid
FDA: Food and Drug Administration
Cd: Cadmium
L-DOPA: Levodopa
REXO: RVG-exosome
DMT: Disease-modifying treatment
PLGA: Polylactic-co-glycolic acid

## References

[1] Asefy Z, Hoseinnejhad S, Ceferov Z. Nanoparticles approaches in neurodegenerative diseases diagnosis and treatment. Neurol Sci. 2021;42:2653–60. 33846881 10.1007/s10072-021-05234-x

[2] Chang CJ, Cravatt BF, Johnson DS, Lim MH. Molecular medicine and neurodegenerative diseases. Chem Soc Rev. 2014;43:6668–71. 25105517 10.1039/C4CS90065K

[3] Palanisamy CP, Pei J, Alugoju P, Anthikapalli NVA, Jayaraman S, Veeraraghavan VP, et al. New strategies of neurodegenerative disease treatment with extracellular vesicles (EVs) derived from mesenchymal stem cells (MSCs). Theranostics. 2023;13:4138–65. 37554286 10.7150/thno.83066 PMC10405853

[4] Wilson DM 3rd, Cookson MR, Van Den Bosch L, Zetterberg H, Holtzman DM, Dewachter I. Hallmarks of neurodegenerative diseases. Cell. 2023;186:693–714. 36803602 10.1016/j.cell.2022.12.032

[5] Erkkinen MG, Kim MO, Geschwind MD. Clinical neurology and epidemiology of the major neurodegenerative diseases. Cold Spring Harb Perspect Biol. 2018;10. 10.1101/cshperspect.a033118 PMC5880171 28716886

[6] Dugger BN, Dickson DW. Pathology of neurodegenerative diseases. Cold Spring Harb Perspect Biol. 2017;9. 10.1101/cshperspect.a028035 PMC5495060 28062563

[7] Prusiner SB. Shattuck lecture–neurodegenerative diseases and prions. N Engl J Med. 2001;344:1516–26. 11357156 10.1056/NEJM200105173442006

[8] Flach M, Leu C, Martinisi A, Skachokova Z, Frank S, Tolnay M, et al. Trans-seeding of Alzheimer-related tau protein by a yeast prion. Alzheimers Dement. 2022;18:2481–92. 35142027 10.1002/alz.12581 PMC10078693

[9] Soto C, Satani N. The intricate mechanisms of neurodegeneration in prion diseases. Trends Mol Med. 2011;17:14–24. 20889378 10.1016/j.molmed.2010.09.001 PMC3056171

[10] Scheckel C, Aguzzi A. Prions, prionoids and protein misfolding disorders. Nat Rev Genet. 2018;19:405–18. 29713012 10.1038/s41576-018-0011-4

[11] Prusiner SB. Molecular biology of prion diseases. Science. 1991;252:1515–22. 1675487 10.1126/science.1675487

[12] Kayed R, Head E, Thompson JL, McIntire TM, Milton SC, Cotman CW, et al. Common structure of soluble amyloid oligomers implies common mechanism of pathogenesis. Science. 2003;300:486–9. 12702875 10.1126/science.1079469

[13] Spires-Jones TL, Hyman BT. The intersection of amyloid beta and tau at synapses in Alzheimer’s disease. Neuron. 2014;82:756–71. 24853936 10.1016/j.neuron.2014.05.004 PMC4135182

[14] Seeley WW, Crawford RK, Zhou J, Miller BL, Greicius MD. Neurodegenerative diseases target large-scale human brain networks. Neuron. 2009;62:42–52. 19376066 10.1016/j.neuron.2009.03.024 PMC2691647

[15] Sudhof TC. The presynaptic active zone. Neuron. 2012;75:11–25. 22794257 10.1016/j.neuron.2012.06.012 PMC3743085

[16] Li S, Sheng ZH. Energy matters: presynaptic metabolism and the maintenance of synaptic transmission. Nat Rev Neurosci. 2022;23:4–22. 34782781 10.1038/s41583-021-00535-8

[17] Cunnane SC, Trushina E, Morland C, Prigione A, Casadesus G, Andrews ZB, et al. Brain energy rescue: an emerging therapeutic concept for neurodegenerative disorders of ageing. Nat Rev Drug Discov. 2020;19:609–33. 32709961 10.1038/s41573-020-0072-x PMC7948516

[18] Armstrong MJ, Okun MS. Diagnosis and treatment of Parkinson disease: a review. JAMA. 2020;323:548–60. 32044947 10.1001/jama.2019.22360

[19] Esmaeili Y, Yarjanli Z, Pakniya F, Bidram E, Los MJ, Eshraghi M, et al. Targeting autophagy, oxidative stress, and ER stress for neurodegenerative disease treatment. J Control Release. 2022;345:147–75. 35248646 10.1016/j.jconrel.2022.03.001

[20] Liaw K, Zhang Z, Kannan S. Neuronanotechnology for brain regeneration. Adv Drug Deliv Rev. 2019;148:3–18. 31668648 10.1016/j.addr.2019.04.004

[21] Bassyouni F, ElHalwany N, Abdel Rehim M, Neyfeh M. Advances and new technologies applied in controlled drug delivery system. Res Chem Intermed. 2013;41:2165–200. 10.1007/s11164-013-1338-2

[22] Campani V, Giarra S, De Rosa G. Lipid-based core-shell nanoparticles: evolution and potentialities in drug delivery. OpenNano. 2018;3:5–17. 10.1016/j.onano.2017.12.001

[23] Vimbela GV, Ngo SM, Fraze C, Yang L, Stout DA. Antibacterial properties and toxicity from metallic nanomaterials. Int J Nanomed. 2017;12:3941–65. 10.2147/IJN.S134526 PMC5449158 28579779

[24] Zhao W, Li A, Zhang A, Zheng Y, Liu J. Recent advances in functional-polymer-decorated transition-metal nanomaterials for bioimaging and cancer therapy. ChemMedChem. 2018;13:2134–49. 30152914 10.1002/cmdc.201800462

[25] Minakshi P, Mohan H, Manjeet, Ravina, Brar B, Shafiq M, et al. Organic polymer and metal nano-particle based composites for improvement of the analytical performance of electrochemical biosensors. Curr Top Med Chem. 2020;20:1029–41. 32148195 10.2174/1568026620666200309092957

[26] Lopes D, Lopes J, Pereira-Silva M, Peixoto D, Rabiee N, Veiga F, et al. Bioengineered exosomal-membrane-camouflaged abiotic nanocarriers: neurodegenerative diseases, tissue engineering and regenerative medicine. Mil Med Res. 2023;10:19. 37101293 10.1186/s40779-023-00453-z PMC10134679

[27] Peng H, Li Y, Ji W, Zhao R, Lu Z, Shen J, et al. Intranasal administration of self-oriented nanocarriers based on therapeutic exosomes for synergistic treatment of Parkinson’s disease. ACS Nano. 2022;16:869–84. 34985280 10.1021/acsnano.1c08473

[28] Lopez-Chaves C, Soto-Alvaredo J, Montes-Bayon M, Bettmer J, Llopis J, Sanchez-Gonzalez C. Gold nanoparticles: distribution, bioaccumulation and toxicity. In vitro and in vivo studies. Nanomedicine. 2018;14:1–12. 28882675 10.1016/j.nano.2017.08.011

[29] Almeida JP, Chen AL, Foster A, Drezek R. In vivo biodistribution of nanoparticles. Nanomedicine (Lond). 2011;6:815–35. 21793674 10.2217/nnm.11.79

[30] Neha D, Momin M, Khan T, Gharat S, Ningthoujam RS, Omri A. Metallic nanoparticles as drug delivery system for the treatment of cancer. Expert Opin Drug Deliv. 2021;18:1261–90. 33793359 10.1080/17425247.2021.1912008

[31] Luk BT, Zhang L. Cell membrane-camouflaged nanoparticles for drug delivery. J Control Release. 2015;220:600–7. 26210440 10.1016/j.jconrel.2015.07.019 PMC4688192

[32] Amini SM, Kharrazi S, Rezayat SM, Gilani K. Radiofrequency electric field hyperthermia with gold nanostructures: role of particle shape and surface chemistry. Artif Cells Nanomed Biotechnol. 2018;46:1452–62. 28891351 10.1080/21691401.2017.1373656

[33] Liang Y, Iqbal Z, Lu J, Wang J, Zhang H, Chen X, et al. Cell-derived nanovesicle-mediated drug delivery to the brain: Principles and strategies for vesicle engineering. Mol Ther. 2023;31:1207–24. 36245129 10.1016/j.ymthe.2022.10.008 PMC10188644

[34] Agrawal M, Prathyusha E, Ahmed H, Dubey SK, Kesharwani P, Singhvi G, et al. Biomaterials in treatment of Alzheimer’s disease. Neurochem Int. 2021;145: 105008. 33684545 10.1016/j.neuint.2021.105008

[35] Ferreira NN, Granja S, Boni FI, Prezotti FG, Ferreira LMB, Cury BSF, et al. Modulating chitosan-PLGA nanoparticle properties to design a co-delivery platform for glioblastoma therapy intended for nose-to-brain route. Drug Deliv Transl Res. 2020;10:1729–47. 32683647 10.1007/s13346-020-00824-2

[36] Liu XG, Zhang L, Lu S, Liu DQ, Zhang LX, Yu XL, et al. Multifunctional superparamagnetic iron oxide nanoparticles conjugated with abeta oligomer-specific SCFV antibody and class a scavenger receptor activator show early diagnostic potentials for Alzheimer’s disease. Int J Nanomedicine. 2020;15:4919–32. 32764925 10.2147/IJN.S240953 PMC7360423

[37] Shah S, Leon L. Structural dynamics, phase behavior, and applications of polyelectrolyte complex micelles. Curr Opin Colloid In. 2021;53.

[38] Agwa MM, Abdelmonsif DA, Khattab SN, Sabra S. Self- assembled lactoferrin-conjugated linoleic acid micelles as an orally active targeted nanoplatform for Alzheimer’s disease. Int J Biol Macromol. 2020;162:246–61. 32531361 10.1016/j.ijbiomac.2020.06.058

[39] Rajkovic O, Potjewyd G, Pinteaux E. Regenerative medicine therapies for targeting neuroinflammation after stroke. Front Neurol. 2018;9:734. 30233484 10.3389/fneur.2018.00734 PMC6129611

[40] Ojeda-Hernandez DD, Canales-Aguirre AA, Matias-Guiu J, Gomez-Pinedo U, Mateos-Diaz JC. Potential of chitosan and its derivatives for biomedical applications in the central nervous system. Front Bioeng Biotechnol. 2020;8:389. 32432095 10.3389/fbioe.2020.00389 PMC7214799

[41] Gong B, Cheng W, Ji W, Chen X, Chu J, Liang W, et al. Hydrogel: a promising new technique for treating Alzheimer’s disease. J Transl Int Med. 2022;10:15–7. 35702184 10.2478/jtim-2022-0008 PMC8997806

[42] Long Y, Xiang Y, Liu S, Zhang Y, Wan J, Ci Z, et al. Macrophage membrane modified baicalin liposomes improve brain targeting for alleviating cerebral ischemia reperfusion injury. Nanomedicine. 2022;43: 102547. 35292367 10.1016/j.nano.2022.102547

[43] Zhong X, Na Y, Yin S, Yan C, Gu J, Zhang N, et al. Cell membrane biomimetic nanoparticles with potential in treatment of Alzheimer's disease. Molecules. 2023;28. 10.3390/molecules28052336 PMC10005336 36903581

[44] Khongkow M, Yata T, Boonrungsiman S, Ruktanonchai UR, Graham D, Namdee K. Surface modification of gold nanoparticles with neuron-targeted exosome for enhanced blood-brain barrier penetration. Sci Rep. 2019;9:8278. 31164665 10.1038/s41598-019-44569-6 PMC6547645

[45] Li C, Zhao Z, Luo Y, Ning T, Liu P, Chen Q, et al. Macrophage-disguised manganese dioxide nanoparticles for neuroprotection by reducing oxidative stress and modulating inflammatory microenvironment in acute ischemic stroke. Adv Sci (Weinh). 2021;8: e2101526. 34436822 10.1002/advs.202101526 PMC8529435

[46] Rahman MA, Wang J, Zhang C, Olah A, Baer E. Novel micro-/nano- porous cellular membranes by forced assembly co-extrusion technology. Eur Polymer J. 2016;83:99–113. 10.1016/j.eurpolymj.2016.08.015

[47] He W, Frueh J, Wu Z, He Q. Leucocyte membrane-coated Janus microcapsules for enhanced photothermal cancer treatment. Langmuir. 2016;32:3637–44. 27023433 10.1021/acs.langmuir.5b04762

[48] Liu Q, Fan T, Zheng Y, Yang SL, Yu Z, Duo Y, et al. Immunogenic exosome-encapsulated black phosphorus nanoparticles as an effective anticancer photo-nanovaccine. Nanoscale. 2020;12:19939–52. 32991664 10.1039/D0NR05953F

[49] Xiong F, Ling X, Chen X, Chen J, Tan J, Cao W, et al. Pursuing specific chemotherapy of orthotopic breast cancer with lung metastasis from docking nanoparticles driven by bioinspired exosomes. Nano Lett. 2019;19:3256–66. 30965009 10.1021/acs.nanolett.9b00824

[50] Liu L, Li Y, Peng H, Liu R, Ji W, Shi Z, et al. Targeted exosome coating gene-chem nanocomplex as "nanoscavenger" for clearing alpha-synuclein and immune activation of Parkinson's disease. Sci Adv. 2020;6. 10.1126/sciadv.aba3967 PMC7732192 33310840

[51] Lu M, Huang Y. Bioinspired exosome-like therapeutics and delivery nanoplatforms. Biomaterials. 2020;242: 119925. 32151860 10.1016/j.biomaterials.2020.119925

[52] Kutchy NA, Ma R, Liu Y, Buch S, Hu G. Extracellular vesicle-mediated delivery of ultrasmall superparamagnetic iron oxide nanoparticles to mice brain. Front Pharmacol. 2022;13: 819516. 35462907 10.3389/fphar.2022.819516 PMC9022024

[53] Jiang Y, Wang R, Wang C, Guo Y, Xu T, Zhang Z, et al. Brain microenvironment responsive and pro-angiogenic extracellular vesicle-hydrogel for promoting neurobehavioral recovery in type 2 diabetic mice after stroke. Adv Healthc Mater. 2022;11: e2201150. 36074801 10.1002/adhm.202201150

[54] Zhang M, Zhang R, Chen H, Zhang X, Zhang Y, Liu H, et al. Injectable supramolecular hybrid hydrogel delivers IL-1beta-stimulated exosomes to target neuroinflammation. ACS Appl Mater Interfaces. 2023;15:6486–98. 36716400 10.1021/acsami.2c19997

[55] Betzer O, Perets N, Angel A, Motiei M, Sadan T, Yadid G, et al. In vivo neuroimaging of exosomes using gold nanoparticles. ACS Nano. 2017;11:10883–93. 28960957 10.1021/acsnano.7b04495

[56] Uppuluri CT, Ravi PR, Dalvi AV. Design, optimization and pharmacokinetic evaluation of Piribedil loaded solid lipid nanoparticles dispersed in nasal in situ gelling system for effective management of Parkinson’s disease. Int J Pharm. 2021;606: 120881. 34273426 10.1016/j.ijpharm.2021.120881

[57] Adnet T, Groo AC, Picard C, Davis A, Corvaisier S, Since M, et al. Pharmacotechnical development of a nasal drug delivery composite nanosystem intended for Alzheimer's disease treatment. Pharmaceutics. 2020;12. 10.3390/pharmaceutics12030251 PMC7151011 32168767

[58] Shah S, Dhawan V, Holm R, Nagarsenker MS, Perrie Y. Liposomes: advancements and innovation in the manufacturing process. Adv Drug Deliv Rev. 2020;154–155:102–22. 32650041 10.1016/j.addr.2020.07.002

[59] Saesoo S, Sathornsumetee S, Anekwiang P, Treetidnipa C, Thuwajit P, Bunthot S, et al. Characterization of liposome-containing SPIONs conjugated with anti-CD20 developed as a novel theranostic agent for central nervous system lymphoma. Colloids Surf B Biointerfaces. 2018;161:497–507. 29128836 10.1016/j.colsurfb.2017.11.003

[60] Shi D, Mi G, Shen Y, Webster TJ. Glioma-targeted dual functionalized thermosensitive Ferri-liposomes for drug delivery through an in vitro blood-brain barrier. Nanoscale. 2019;11:15057–71. 31369016 10.1039/C9NR03931G

[61] Pooja D, Tunki L, Kulhari H, Reddy BB, Sistla R. Optimization of solid lipid nanoparticles prepared by a single emulsification-solvent evaporation method. Data Brief. 2016;6:15–9. 26759823 10.1016/j.dib.2015.11.038 PMC4683325

[62] Gomes MJ, Fernandes C, Martins S, Borges F, Sarmento B. Tailoring lipid and polymeric nanoparticles as siRNA carriers towards the blood-brain barrier - from targeting to safe administration. J Neuroimmune Pharmacol. 2017;12:107–19. 27209050 10.1007/s11481-016-9685-6

[63] Stoessl AJ. Neuroimaging in the early diagnosis of neurodegenerative disease. Transl Neurodegener. 2012;1:5. 23211024 10.1186/2047-9158-1-5 PMC3506998

[64] Padmanabhan P, Kumar A, Kumar S, Chaudhary RK, Gulyas B. Nanoparticles in practice for molecular-imaging applications: an overview. Acta Biomater. 2016;41:1–16. 27265153 10.1016/j.actbio.2016.06.003

[65] Thery C, Zitvogel L, Amigorena S. Exosomes: composition, biogenesis and function. Nat Rev Immunol. 2002;2:569–79. 12154376 10.1038/nri855

[66] Hessvik NP, Llorente A. Current knowledge on exosome biogenesis and release. Cell Mol Life Sci. 2018;75:193–208. 28733901 10.1007/s00018-017-2595-9 PMC5756260

[67] Elliott RO, He M. Unlocking the power of exosomes for crossing biological barriers in drug delivery. Pharmaceutics. 2021;13. 10.3390/pharmaceutics13010122 PMC7835896 33477972

[68] Barjesteh T, Mansur S, Bao Y. Inorganic nanoparticle-loaded exosomes for biomedical applications. Molecules. 2021;26. 10.3390/molecules26041135 PMC7924372 33672706

[69] Perets N, Betzer O, Shapira R, Brenstein S, Angel A, Sadan T, et al. Golden exosomes selectively target brain pathologies in neurodegenerative and neurodevelopmental disorders. Nano Lett. 2019;19:3422–31. 30761901 10.1021/acs.nanolett.8b04148

[70] Wang Z, Gonzalez KM, Cordova LE, Lu J. Nanotechnology-empowered therapeutics targeting neurodegenerative diseases. Wiley Interdiscip Rev Nanomed Nanobiotechnol. 2023;15: e1907. 37248794 10.1002/wnan.1907 PMC10525015

[71] Lee HJ, Yoon YS, Lee SJ. Molecular mechanisms of cellular senescence in neurodegenerative diseases. J Mol Biol. 2023;435: 168114. 37085010 10.1016/j.jmb.2023.168114

[72] Feng L, Dou C, Xia Y, Li B, Zhao M, Yu P, et al. Neutrophil-like cell-membrane-coated nanozyme therapy for ischemic brain damage and long-term neurological functional recovery. ACS Nano. 2021;15:2263–80. 33426885 10.1021/acsnano.0c07973

[73] Han Y, Chu X, Cui L, Fu S, Gao C, Li Y, et al. Neuronal mitochondria-targeted therapy for Alzheimer’s disease by systemic delivery of resveratrol using dual-modified novel biomimetic nanosystems. Drug Deliv. 2020;27:502–18. 32228100 10.1080/10717544.2020.1745328 PMC7170363

[74] Han Y, Gao C, Wang H, Sun J, Liang M, Feng Y, et al. Macrophage membrane-coated nanocarriers Co-Modified by RVG29 and TPP improve brain neuronal mitochondria-targeting and therapeutic efficacy in Alzheimer’s disease mice. Bioact Mater. 2021;6:529–42. 32995678 10.1016/j.bioactmat.2020.08.017 PMC7492821

[75] Collaborators GBDD. Global, regional, and national burden of Alzheimer’s disease and other dementias, 1990–2016: a systematic analysis for the Global Burden of Disease Study 2016. Lancet Neurol. 2019;18:88–106. 30497964 10.1016/S1474-4422(18)30403-4 PMC6291454

[76] Peng Y, Jin H, Xue YH, Chen Q, Yao SY, Du MQ, et al. Current and future therapeutic strategies for Alzheimer’s disease: an overview of drug development bottlenecks. Front Aging Neurosci. 2023;15:1206572. 37600514 10.3389/fnagi.2023.1206572 PMC10438465

[77] Guo X, Yan L, Zhang D, Zhao Y. Passive immunotherapy for Alzheimer’s disease. Ageing Res Rev. 2024;94: 102192. 38219962 10.1016/j.arr.2024.102192

[78] Jucker M, Walker LC. Alzheimer’s disease: from immunotherapy to immunoprevention. Cell. 2023;186:4260–70. 37729908 10.1016/j.cell.2023.08.021 PMC10578497

[79] van Dyck CH, Swanson CJ, Aisen P, Bateman RJ, Chen C, Gee M, et al. Lecanemab in early Alzheimer’s disease. N Engl J Med. 2023;388:9–21. 36449413 10.1056/NEJMoa2212948

[80] Tang D, Wang Y, Wijaya A, Liu B, Maruf A, Wang J, et al. ROS-responsive biomimetic nanoparticles for potential application in targeted anti-atherosclerosis. Regen Biomater. 2021;8:rbab033. 34285811 10.1093/rb/rbab033 PMC8286794

[81] Cunha S, Swedrowska M, Bellahnid Y, Xu Z, Sousa Lobo JM, Forbes B, et al. Thermosensitive in situ hydrogels of rivastigmine-loaded lipid-based nanosystems for nose-to-brain delivery: characterisation, biocompatibility, and drug deposition studies. Int J Pharm. 2022;620: 121720. 35413397 10.1016/j.ijpharm.2022.121720

[82] Zaazaa AM, Abd El-Motelp BA, Ali NA, Youssef AM, Sayed MA, Mohamed SH. Stem cell-derived exosomes and copper sulfide nanoparticles attenuate the progression of neurodegenerative disorders induced by cadmium in rats. Heliyon. 2022;8: e08622. 35028441 10.1016/j.heliyon.2021.e08622 PMC8741450

[83] Kakkar AK, Singh H, Medhi B. Old wines in new bottles: repurposing opportunities for Parkinson’s disease. Eur J Pharmacol. 2018;830:115–27. 29689247 10.1016/j.ejphar.2018.04.023

[84] Agostini F, Masato A, Bubacco L, Bisaglia M. Metformin repurposing for parkinson disease therapy: opportunities and challenges. Int J Mol Sci. 2021;23. 10.3390/ijms23010398 PMC8745385 35008822

[85] Dobson R, Giovannoni G. Multiple sclerosis - a review. Eur J Neurol. 2019;26:27–40. 30300457 10.1111/ene.13819

[86] Klineova S, Lublin FD. Clinical course of multiple sclerosis. Cold Spring Harb Perspect Med. 2018;8. 10.1101/cshperspect.a028928 PMC6120692 29358317

[87] Montalban X, Gold R, Thompson AJ, Otero-Romero S, Amato MP, Chandraratna D, et al. ECTRIMS/EAN guideline on the pharmacological treatment of people with multiple sclerosis. Mult Scler. 2018;24:96–120. 29353550 10.1177/1352458517751049

[88] Hauser SL, Cree BAC. Treatment of multiple sclerosis: a review. Am J Med. 2020;133(1380–90): e2. 10.1016/j.amjmed.2020.05.049 PMC7704606 32682869

[89] Dangond F, Donnelly A, Hohlfeld R, Lubetzki C, Kohlhaas S, Leocani L, et al. Facing the urgency of therapies for progressive MS—a Progressive MS Alliance proposal. Nat Rev Neurol. 2021;17:185–92. 33483719 10.1038/s41582-020-00446-9

[90] Ferreira H, Amorim D, Lima AC, Pirraco RP, Costa-Pinto AR, Almeida R, et al. A biocompatible and injectable hydrogel to boost the efficacy of stem cells in neurodegenerative diseases treatment. Life Sci. 2021;287: 120108. 34717909 10.1016/j.lfs.2021.120108

[91] Valenza M, Birolini G, Cattaneo E. The translational potential of cholesterol-based therapies for neurological disease. Nat Rev Neurol. 2023;19:583–98. 37644213 10.1038/s41582-023-00864-5

[92] Zuccato C, Valenza M, Cattaneo E. Molecular mechanisms and potential therapeutical targets in Huntington’s disease. Physiol Rev. 2010;90:905–81. 20664076 10.1152/physrev.00041.2009

[93] Tosi G, Fano RA, Bondioli L, Badiali L, Benassi R, Rivasi F, et al. Investigation on mechanisms of glycopeptide nanoparticles for drug delivery across the blood-brain barrier. Nanomedicine (Lond). 2011;6:423–36. 21542682 10.2217/nnm.11.11

[94] Belletti D, Grabrucker AM, Pederzoli F, Menrath I, Vandelli MA, Tosi G, et al. Hybrid nanoparticles as a new technological approach to enhance the delivery of cholesterol into the brain. Int J Pharm. 2018;543:300–10. 29608954 10.1016/j.ijpharm.2018.03.061

[95] Duan S, Yang J, Cui Z, Li J, Zheng H, Zhao T, et al. Seed amplification assay of nasal swab extracts for accurate and non-invasive molecular diagnosis of neurodegenerative diseases. Transl Neurodegener. 2023;12:13. 36922862 10.1186/s40035-023-00345-1 PMC10017346

